# Cerebrospinal Fluid Cortisol and Dehydroepiandrosterone Sulfate, Alzheimer’s Disease Pathology, and Cognitive Decline

**DOI:** 10.3389/fnagi.2022.892754

**Published:** 2022-07-07

**Authors:** Sami Ouanes, Christopher Clark, Jonas Richiardi, Bénédicte Maréchal, Piotr Lewczuk, Johannes Kornhuber, Clemens Kirschbaum, Julius Popp

**Affiliations:** ^1^Service of Old Age Psychiatry, Department of Psychiatry, Lausanne University Hospital, Lausanne, Switzerland; ^2^Department of Psychiatry, Hamad Medical Corporation, Doha, Qatar; ^3^Centre for Gerontopsychiatric Medicine, Geriatric Psychiatry, University Hospital of Psychiatry Zürich, Zurich, Switzerland; ^4^Department of Radiology, Lausanne University Hospital, Lausanne, Switzerland; ^5^Department of Psychiatry and Psychotherapy, University Hospital, Friedrich-Alexander-University Erlangen-Nuremberg, Erlangen, Germany; ^6^Chair of Biopsychology, Technische Universität Dresden, Andreas-Schubert-Bau, Dresden, Germany

**Keywords:** cortisol, DHEAS, cognitive decline, Alzheimer’s disease, neurodegeneration, cerebrospinal fluid

## Abstract

**Introduction:**

Elevated cortisol levels have been reported in Alzheimer’s disease (AD) and may accelerate the development of brain pathology and cognitive decline. Dehydroepiandrosterone sulfate (DHEAS) has anti-glucocorticoid effects and it may be involved in the AD pathophysiology.

**Objectives:**

To investigate associations of cerebrospinal fluid (CSF) cortisol and DHEAS levels with (1) cognitive performance at baseline; (2) CSF biomarkers of amyloid pathology (as assessed by CSF Aβ levels), neuronal injury (as assessed by CSF tau), and tau hyperphosphorylation (as assessed by CSF p-tau); (3) regional brain volumes; and (4) clinical disease progression.

**Materials and Methods:**

Individuals between 49 and 88 years (*n* = 145) with mild cognitive impairment or dementia or with normal cognition were included. Clinical scores, AD biomarkers, brain MRI volumetry along with CSF cortisol and DHEAS were obtained at baseline. Cognitive and functional performance was re-assessed at 18 and 36 months from baseline. We also assessed the following covariates: apolipoprotein E (APOE) genotype, BMI, and education. We used linear regression and mixed models to address associations of interest.

**Results:**

Higher CSF cortisol was associated with poorer global cognitive performance and higher disease severity at baseline. Cortisol and cortisol/DHEAS ratio were positively associated with tau and p-tau CSF levels, and negatively associated with the amygdala and insula volumes at baseline. Higher CSF cortisol predicted more pronounced cognitive decline and clinical disease progression over 36 months. Higher CSF DHEAS predicted more pronounced disease progression over 36 months.

**Conclusion:**

Increased cortisol in the CNS is associated with tau pathology and neurodegeneration, and with decreased insula and amygdala volume. Both CSF cortisol and DHEAS levels predict faster clinical disease progression. These results have implications for the identification of patients at risk of rapid decline as well as for the development of interventions targeting both neurodegeneration and clinical manifestations of AD.

## Introduction

Different steroids including cortisol, dehydroepiandrosterone (DHEA) and its sulfate ester (DHEAS) have been shown to exert crucial actions on the brain, translating into effects on brain aging, neurocognition, and the pathogenesis and course of neurocognitive disorders, in particular Alzheimer’s disease (AD) (Maninger et al., 2009; Dong and Zheng, 2012; Ouanes and Popp, 2019).

Cortisol has been associated with impeding normal amyloid β (Aβ) and tau processing in the brain, exacerbating Aβ and tau toxicity, promoting oxidative stress and neurodegeneration, inducing synaptic dysfunction, and reducing dendritic plasticity in the hippocampus and prefrontal cortex in animal models (Ouanes and Popp, 2019). Higher cortisol levels have been reported in patients with clinically diagnosed AD dementia or mild cognitive impairment (MCI) compared to controls, in plasma, saliva, urine, and cerebrospinal fluid (CSF) (Zheng et al., 2020). Previous studies also found that elevated cortisol levels were associated with more pronounced cognitive impairment in patients with AD dementia (Zverova et al., 2013; Pena-Bautista et al., 2019), as well as with faster cognitive decline in cognitively healthy older individuals (Pietrzak et al., 2017) and in patients with MCI or dementia of AD type (Huang et al., 2009; Popp et al., 2015).

The role of DHEAS on the brain is complex as it has both direct and indirect effects following conversion into testosterone and estrogens (Maninger et al., 2009). DHEAS is involved in promoting neuroprotection and neurogenesis including mechanisms such as stimulation of neurite growth and inhibition of neuronal apoptosis. DHEAS also exhibits anti-glucocorticoid effects (Maninger et al., 2009; Hampl and Bicikova, 2010), and has been found to have antidepressant, anxiolytic and cognitive enhancing effects (Dong and Zheng, 2012). DHEAS levels in both plasma and CSF have been reported to be lower in patients with AD dementia (Kim et al., 2003; Aldred and Mecocci, 2010).

Given that DHEAS and cortisol often display antagonistic actions in the brain, cortisol/DHEAS ratio has been used to address interrelated effects. Plasma cortisol/DHEAS ratio was reported to be elevated in patients with dementia (De Bruin et al., 2002; Magri et al., 2006), and specifically in patients with AD dementia (Armanini et al., 2003), compared to controls.

Despite the growing body of evidence that steroids, in particular cortisol and DHEAS, might be incriminated in the etiopathogenesis of AD and might be considered potential therapeutic targets, there have been very few studies that assessed both cortisol and DHEAS levels in human subjects with MCI or AD type dementia (Maninger et al., 2009; Ouanes and Popp, 2019). While assessments of salivary or plasma levels are more practical, CSF levels are more tightly linked to the effects on the brain, informing more accurately about the concentrations to which the brain structures are exposed, especially given that there is evidence of possible synthesis of these steroids in the brain (Maninger et al., 2009; Popp et al., 2009, 2015). Very few studies have addressed the relationships between CSF cortisol and AD pathology in humans, and, to the best of our knowledge, no previous prospective study examined the links between CSF DHEAS and markers of AD pathology, and cognitive decline over time. DHEAS in AD has been understudied even though its neuroprotective properties and its potential beneficial effects on cognition and neurodegeneration may translate into clinical and therapeutic implications (Strac et al., 2020).

In the present paper, we aimed to investigate whether CSF Cortisol and DHEAS were associated with (1) cognitive and functional performance at baseline; (2) with amyloid pathology (as assessed by CSF Aβ levels), neuronal injury (as assessed by CSF tau), and tau hyperphosphorylation (as assessed by CSF phosphorylated tau or p-tau); (3) regional brain volumetry; and (4) with clinical disease progression at 18 then at 36 months from baseline.

## Materials and Methods

### Participants

We recruited 145 community-dwelling individuals aged between 49 and 88 years in an observational study on biomarkers of cognitive decline and AD conducted at the Department of Psychiatry and the Department of Clinical Neurosciences, Lausanne University Hospital, Switzerland.

The study participants with cognitive impairment (*n* = 93) were recruited among outpatients with cognitive impairment (MCI or mild dementia) referred to the Memory Clinics, Department of Psychiatry and the Department Clinical Neurosciences, Lausanne University Hospital for investigation of cognitive complaints. The diagnosis of MCI or of dementia was based on neuropsychological and clinical evaluations according to published criteria (Mckhann et al., 2011); and was made by a consensus conference of senior physicians and neuropsychologists before inclusion into the study. All subjects in this group had a Clinical Dementia Rating Scale (CDR) (Morris, 1993) score ≥ 0.5 based on the clinical and neuropsychological examination and considering informant questionnaires on the patient’s health and activities of daily living (ADLs), as described elsewhere (Mathys et al., 2017). Participants with a diagnosis of MCI or mild dementia were considered together, in accordance with recent concepts of AD considering the disease as a biological continuum of developing cerebral pathology across clinical stages (Albert et al., 2011). Cognitively healthy individuals (*n* = 52) were recruited through journal announcements and word of mouth. They underwent the same clinical and neuropsychological examination as the cognitively impaired participants. They had no history or clinical signs of cognitive decline and the absence of cognitive impairment was confirmed by a multi-domain cognitive and functional neuropsychological assessment (as detailed below) that allowed to confirm that their CDR score was 0. Clinical examination and the Hospital Anxiety and Depression Scale (Zigmond and Snaith, 1983) were used in all participants to exclude the presence of clinically relevant depression or anxiety symptoms. We excluded subjects with any concomitant neurological, psychiatric or somatic comorbidity, or current medication that could affect cognition at baseline.

At follow-up visits 18 and 36 months from baseline, cognitive and functional performance was assessed using the same methods as at baseline.

For the longitudinal findings, we chose to analyze all participants within a single cohort, consistently with the hypothesis that pre-clinical stages, MCI, and dementia are part of the same continuum of neurodegeneration (Albert et al., 2011). Indeed participants who were “cognitively healthy” at baseline may transition to MCI or dementia by the end of the study.

### Cognitive Assessment

At baseline and at each follow-up visit (18 then 36 months), a detailed neuropsychological evaluation was completed and information on the participants’ daily life activities was collected by a trained psychologist blinded to the investigated biological markers, as previously described (Mathys et al., 2017).

The neuropsychological assessment included:

• Mini Mental State Examination (MMSE) for global cognitive performance.
• Grober and Buschke Double Memory Test (Buschke et al., 1997) for *episodic memory.*
• DO40 picture-naming test (Deloche and Hannequin, 1997), the phonemic and the semantic fluency tasks for *verbal fluency.*
• Stroop Test for *executive functions.*
• Figures from the Consortium to Establish a Registry for Alzheimer’s Disease (CERAD) neuropsychological test battery (Morris et al., 1988) for *visuospatial construction.*

The functional assessment included the Katz Index of Independence in ADLs (Katz et al., 1963) for basic ADLs and Lawton Instrumental ADL Scale for instrumental ADLs (Lawton and Brody, 1969).

The questionnaires and tools above were used to determine the overall cognitive and functional status by the CDR scale. CDR is widely used for the clinical staging of cognitive impairment (Morris, 1993). The CDR sum of boxes score (CDR-SB) was also calculated.

In addition to the CDR-SB scores derived from the detailed neuropsychological evaluation and used as an outcome variable to assess cognitive and functional decline, MMSE scores were also used as an outcome variable. MMSE is a measure of global cognition, not allowing for comprehensive evaluation of subdomains of cognition. It is also of limited value in cognitively normal people because of floor effect. However, MMSE score change in time is widely used in clinical practice as well as in research to assess global disease progression (Schmidt et al., 2011).

### Cerebrospinal Fluid Biomarkers

Ten to twelve mL of CSF were collected by lumbar puncture between 8 and 9 AM after overnight fasting. The CSF samples were spun down at 4°C, immediately aliquoted, and snap frozen at −80°C until assay (Popp et al., 2017). Blood samples were collected 15–30 min later.

Cerebrospinal fluid Amyloid-β_*1–42*_ (Aβ_*1–42*_), Aβ_*1–40*_, tau and p-tau were measured using commercially available enzyme-linked immunosorbent assay kits (Fujirebio, Gent, Belgium). In addition, the levels of Aβ_*1–42*_ and Aβ_*1–40*_ were measured with immunoassays from IBL International (Hamburg, Germany) according to the manufacturer’s protocols.

We used the p-tau/Aβ_*1–42*_ ratio with a cut-off of 0.0779 to define the presence/absence of an AD CSF profile, previously determined as the center cutoff indicating the concomitant presence of amyloid and tau pathology (Popp et al., 2017).

Cerebrospinal fluid cortisol and DHEAS concentrations were measured using commercially available chemiluminescence immunoassay with high sensitivity (IBL International, Hamburg, Germany). Since DHEAS is a robust measure of DHEA production, we focused on DHEAS rather than DHEA in this study (Baulieu and Robel, 1996). The intra and interassay coefficients for cortisol and DHEAS were below 6 and 9%, respectively.

### Neuroimaging

An MRI of the brain was obtained at baseline for each of the participants.

Gray matter (GM) and white matter (WM) regions of interest (ROIs) were defined and segmented using the MorphoBox prototype as described in Schmitter et al. (2015): ROIs were segmented manually by a neurologist and corrected by two neuroradiologists, using a single-subject T1-weighted template of a 64-year-old female with no alcohol dependence or central nervous system disorders scanned, at 3T using the ADNI-2 protocol. After B1 receive correction and gradient distortion correction, the template is non-rigidly registered to the input image. Volumes of 45 regions are estimated by combining atlas-free tissue classification and atlas-based segmentation on the bias-field corrected and skull-stripped input image.

Here we used regional volumetric data normalized by total intracranial volume (defined as the sum of GM, WM, and CSF) to determine relative volumes of ROIs.

Here we used regional volumetric data normalized by total intracranial volume (defined as the sum of GM, WM, and CSF) to determine relative volumes of ROIs.

### Other Variables

To evaluate possible effects of the *apolipoprotein E (APOE)* genotype on the addressed relationships, leukocyte genomic DNA was isolated from EDTA blood with the Qiagen blood isolation kit (Qiagen, Hilden, Germany) and the *APOE* genotype was determined as previously described (Mathys et al., 2017). We split participants into two groups: one having at least one APOEε4 allele (APOEε4 carriers), and the other having no APOEε4 allele (APOEε4 non-carriers).

*Body Mass Index (BMI)* was also considered as a covariate because cortisol levels have been shown to depend on the BMI and BMI change has been associated with AD pathology (Mathys et al., 2017; Kivimaki et al., 2018).

*Education* was assessed using the number of years that the individual completed.

Other standardized measures like for depressive and anxiety symptoms were not available.

### Standard Protocol Approvals, Registrations, and Patient Consents

The study was conducted in accordance with applicable laws and regulations, including the International Conference on Harmonization, Guideline for Good Clinical Practice and the ethical principles that have their origins in the Declaration of Helsinki (World Medical Association, 2013). The local ethical committee approved this study (No. 171/2013), and all participants or their legal representatives provided written informed consent.

### Statistical Analysis

Statistical analysis was performed using SPSS v26.0 (IBM Corp., Armonk, NY, United States).

#### Descriptive Statistics

For categorical variables, we calculated absolute and relative frequencies. For continuous variables, we calculated means and standard deviations. Median values were also determined whenever the distribution was not gaussian (as per the Shapiro–Wilk test).

For each of the analyses below, we chose the following covariates:

– For associations only involving biological measures (CSF cortisol, CSF AD biomarkers, brain volumes), we used age and sex as covariates.
– For associations also involving cognitive performance, we used age, sex, years of education, BMI, and APOEε4 status as covariates. Apart from the level of education which obviously influences the results of most cognitive tests, we added APOE and BMI as covariates because they were shown to influence cognitive performance both in AD and in normal aging (Small et al., 2004; Michaud et al., 2018).

#### Associations Between Cerebrospinal Fluid Cortisol and Dehydroepiandrosterone Sulfate and Baseline Cognitive and Functional Performance

We used the independent samples *t*-test to compare CSF cortisol, CSF DHEAS, and CSF cortisol/DHEAS ratio between participants with baseline cognitive impairment (CDR score > 0) and those without (CDR = 0).

We also constructed separate multiple linear regression models with CSF cortisol and DHEAS as independent variables, and with the baseline MMSE score and CDR-SB, each as a dependent variable in separate analysis, controlling for age, sex, years of education, BMI, and APOEε4 status.

#### Associations of Cerebrospinal Fluid Cortisol and Dehydroepiandrosterone Sulfate With Cerebrospinal Fluid Biomarkers of Alzheimer’s Disease

We compared CSF cortisol, CSF DHEAS, and CSF cortisol/DHEAS ratio between participants with an AD CSF profile (p-tau/Aβ_*1–42*_ ratio > 0.0779) and those without, before then after stratification by the presence of baseline cognitive impairment. We used the independent samples *t*-test whenever valid (sample size ≥ 30 in each group), and Mann–Whitney *U*-test otherwise.

Since most of the variables did not follow a normal distribution, we used Spearman’s non-parametric correlations to examine the associations between CSF cortisol, DHEAS and cortisol/DHEAS ratio and the CSF AD biomarkers (Aβ_*1–42*_, Aβ_*1–42*_/Aβ_*1–40*_ ratio, tau, and p-tau), controlling for age and sex.

#### Associations Between Cerebrospinal Fluid Cortisol and Dehydroepiandrosterone Sulfate and Brain Volumes

We used non-parametric correlations to examine the associations between CSF cortisol, DHEAS and cortisol/DHEAS ratio and the volumes of the following brain structures: GM, WM, frontal GM, frontal WM, parietal GM, parietal WM, temporal GM, temporal WM, occipital GM, occipital WM, hippocampus, amygdala, insula, striatum, and thalamus.

#### Associations Between Cerebrospinal Fluid Cortisol and Dehydroepiandrosterone Sulfate and Cognitive and Functional Decline at 18 and 36 Months From Baseline

We constructed mixed design repeated measures ANOVAs with:

– Clinical Dementia Rating Scale sum of boxes scores (at baseline, at 18 months, and at 36 months) as within-subject variables in a first model then MMSE scores (at baseline, at 18 months, and at 36 months) in a second model
– with sex and APOEε4 as between-subject factors
– and with age, years of education, and CSF cortisol and CSF DHEAS as covariates in a first model, then with age, years of education, and CSF cortisol/DHEAS ratio as covariates in a second model.

Global analyses were followed by between-subject and within-subject analyses to highlight significant interactions. Greenhouse–Geisser correction was applied whenever the sphericity assumption was not fulfilled. Effect sizes were determined using partial eta squares (η^2^).

For all statistical tests, the alpha value was set at 0.05. For multiple comparisons, *p*-values were adjusted according to Holm–Bonferroni’s method.

## Results

### Baseline Characteristics

Baseline sample characteristics of the sample and subgroups defined according to baseline cognitive status are given in Table 1.

**TABLE 1:** General characteristics of the studied sample at baseline.

	Total sample	Participants with CI	Participants with normal cognition	*p*
*n*	145	93	52	
Age, years (m ± SD)	71.2 ± 8.1	74.1 ± 6.7	65.9 ± 7.8	0.001
Gender, *n* (%) women	89 (61.4)	55 (59.1)	34 (65.4)	0.459
Education level*, *n* (%) higher education	43 (30.5)	25 (28.1)	18 (34.6)	0.268
BMI*, Kg/m^2^ (m ± SD)	24.9 ± 4.1	24.8 ± 3.9	25.2 ± 4.4	0.661
APOEε 4*, *n* (%) carriers	48 (33.1)	39 (45.9)	9 (17.6)	0.001
CDR-SB (m ± SD) (0–18)	1.5 ± 2.1	2.3 ± 2.2	0.0 ± 0.1	<0.001
MMSE score (0–30) (m ± SD)	26.4 ± 3.7	25.1 ± 4.0	28.5 ± 1.2	<0.001
**CSF AD biomarkers**				
Aβ_1−42_, in pg/mL (m ± SD)	817.7 ± 284.6	727.3 ± 274.6	979.4 ± 226.0	<0.001
Aβ_1−42⁣/_ *A*β_1−40_ ratio* (m ± SD)	0.055 ± 0.022	0.047 ± 0.017	0.068 ± 0.022	<0.001
tau, in pg/mL (m ± SD)	417.1 ± 308.5	510.1 ± 341.5	250.8 ± 120.4	<0.001
p-tau, in pg/mL (m ± SD)	64.4 ± 36.1	73.3 ± 40.9	48.4 ± 16.1	<0.001
p-tau_/_Aβ_1−42_ ratio (m ± SD)	0.10 ± 0.09	0.12 ± 0.10	0.05 ± 0.02	<0.001
CSF AD biomarker profile, n (%)	59 (40.7)	55 (59.1)	4 (7.7)	<0.001
CSF Cortisol, in ng/mL (m ± SD)	35.94 ± 17.31	39.92 ± 18.10	28.82 ± 13.20	<0.001
CSF DHEAS, in ng/mL (m ± SD)	0.99 ± 0.48	0.99 ± 0.49	0.98 ± 0.47	0.874
CSF Cortisol/DHEAS ratio (m ± SD)	48.44 ± 47.41	54.90 ± 54.92	36.88 ± 26.31	0.009

AD, Alzheimer’s disease; APOE, apolipoprotein E; BMI, body mass index; CDR, clinical dementia rating; CDR-SB, clinical dementia rating sum of boxes; CI, cognitive impairment; CSF, cerebrospinal fluid; DHEAS, dehydroepiandrosterone sulfate; m, mean; MMSE, mini mental state examination; p-tau, hyperphosphorylated tau; SD, standard deviation; tau, total tau. *Missing data: 7 (4.8%) for education level, 11 (7.6%) for BMI, 9 (6.2%) for APOE, 8 (5.5%) for Aβ_1–42_/Aβ_1–40_ ratio. No missing data for the other variables.

Participants with cognitive impairment at baseline (*n* = 93) did not differ from participants without cognitive impairment (*n* = 52) regarding sex, education level, and BMI. Participants with cognitive impairment were significantly older, and more likely to be APOEε4 carriers than participants without cognitive impairment (Table 1).

No correlation was found between CSF cortisol and DHEAS levels.

### Associations Between Cerebrospinal Fluid Cortisol and Dehydroepiandrosterone Sulfate and Baseline Cognitive and Functional Performance

Higher CSF cortisol was associated with poorer baseline cognitive performance as measured by the MMSE score (*B* = −0.038, 95%CI [−0.074; −0.002]; *p* = 0.039; *r* = −0.191), and with poorer baseline cognitive and functional performance as measured by the CDR-SB (*B* = 0.0024 [0.002; 0.045]; *p* = 0.030; *r* = 0.196), after controlling for age, sex, years of education, BMI, and APOEε4 status.

Neither CSF DHEAS nor CSF cortisol/DHEAS ratio were significantly associated with baseline MMSE or CDR-SB.

### Associations Between Baseline Cerebrospinal Fluid Cortisol and Dehydroepiandrosterone Sulfate and Baseline Cerebrospinal Fluid Biomarkers of Alzheimer’s Disease

**FIGURE 1:**
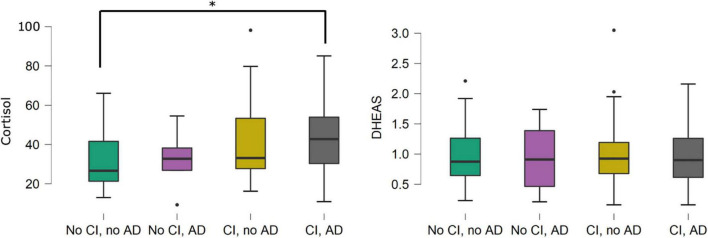
Boxplots of cerebrospinal fluid cortisol and dehydroepiandrosterone sulfate levels in participants with/without cognitive impairment, and with/without a cerebrospinal fluid profile suggestive of Alzheimer’s disease. *p = 0.001. Cerebrospinal fluid cortisol was significantly higher in the AD CI group compared to the no CI, no AD group. The other inter-group differences were not statistically significant. There were no statistically significant differences in cerebrospinal fluid dehydroepiandrosterone sulfate levels between the four groups defined by CI and AD status. Associations shown in this figure were not adjusted for covariates. AD, cerebrospinal fluid profile suggestive of Alzheimer’s disease; CI, cognitive impairment; DHEAS, dehydroepiandrosterone sulfate.

At baseline, CSF cortisol was significantly higher in participants with an AD CSF profile than in those without (42.3 vs. 32.9 ng/mL, *p* = 0.011), while CSF DHEAS and CSF cortisol/DHEAS ratio did not differ significantly between groups. When comparing groups with/without cognitive impairment, and those with/without CSF AD profile, CSF cortisol was significantly higher in patients with cognitive impairment and CSF AD profile than in patients without cognitive impairment and without CSF AD profile (41.1 vs. 29.8 ng/mL, *p* = 0.001). No other differences between subgroups were found (Figure 1).

We observed significant positive correlations between CSF cortisol and CSF cortisol/DHEAS ratio and tau, and p-tau levels at baseline. No significant correlations between CSF DHEAS or CSF cortisol/DHEAS ratio and any of the CSF AD biomarkers was found.

**FIGURE 2:**
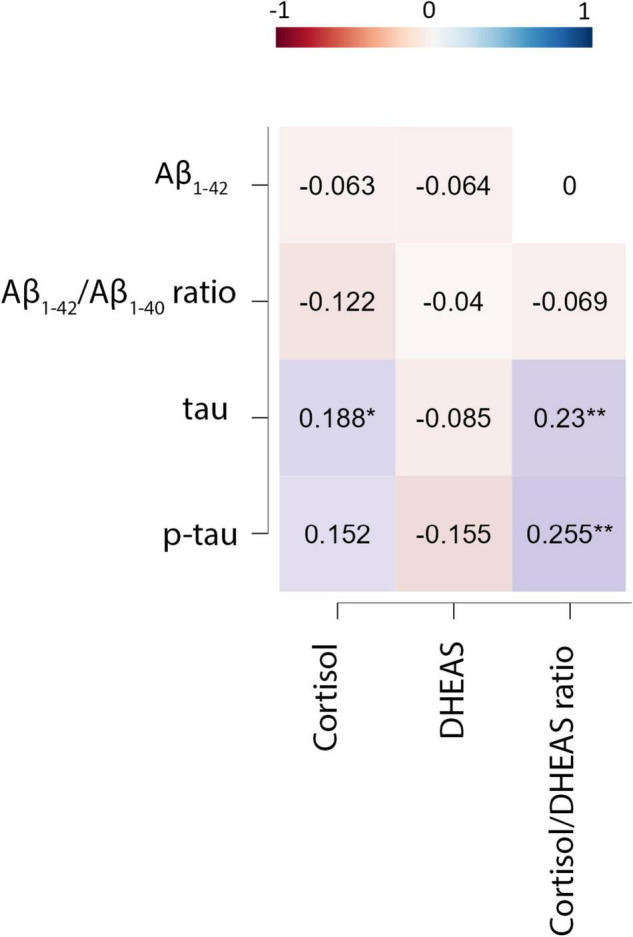
Partial Spearman’s correlations between cerebrospinal fluid cortisol and DHEAS and cerebrospinal biomarkers of Alzheimer’s disease (adjusted for age and sex). CSF, cerebrospinal fluid; DHEAS, dehydroepiandrosterone sulfate; tau, total tau; p-tau, hyperphosphorylated tau; p’, *p*-values adjusted according to Holm–Bonferroni’s method. *p < 0.05; **p < 0.001.

After controlling for age and sex, CSF cortisol remained associated with CSF tau, and cortisol/DHEAS ratio remained associated with CSF tau and p-tau levels (Figure 2).

### Associations Between Baseline Cerebrospinal Fluid Cortisol and Dehydroepiandrosterone Sulfate and Baseline Brain Volumes

**FIGURE 3:**
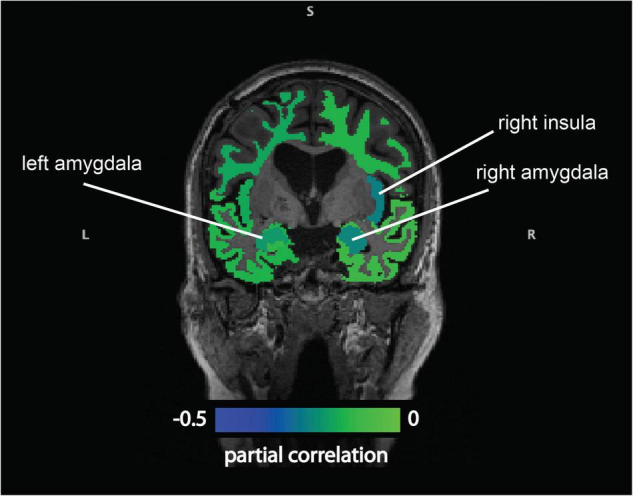
Partial Spearman’s correlations between cerebrospinal fluid cortisol and regional brain volumes (adjusted for age and sex). There were significant negative partial correlations between baseline cerebrospinal fluid cortisol levels and the volumes of the amygdala, and the insula (adjusted for sex and age).

We found significant negative correlations between baseline CSF cortisol levels and volumes of the frontal WM, the temporal GM, the amygdala, and the insula (Supplementary Table 1) at baseline. After controlling for age and sex, CSF cortisol remained negatively associated with the volumes of the amygdala, and the insula (*B* = −0.28 [−0.50; −0.007], *p* = 0.010, *r* = −0.241; and *B* = −0.102 [−0.183; −0.021], *p* = 0.014, *r* = −0.231, respectively) (Figure 3).

No significant associations were found between CSF DHEAS or cortisol/DHEAS ratio and any of the brain volumes.

When we analyzed the associations between baseline CSF cortisol and DHEAS and baseline brain volumes in each group (with vs. without CI at baseline) separately, no significant associations were observed.

### Associations Between Cerebrospinal Fluid Cortisol and Dehydroepiandrosterone Sulfate and Cognitive and Functional Decline at 18 and 36 Months From Baseline

Mixed design repeated measures ANOVAs found significant interactions between CDR-SB changes over time and CSF cortisol (*F*_(1.6, 14.2)_ = 4.5, *p* = 0.019, η^2^ = 0.045) and CSF DHEAS (*F*_(1.6, 11.6)_ = 3.7, *p* = 0.037, η^2^ = 0.037).

When we stratified the analysis by the presence of baseline CI, we found that the interactions between CDR-SB changes over time and CSF cortisol on the one hand and CSF DHEAS on the other hand were not significant in any of the groups.

Similarly, we found significant interactions between MMSE score changes over time and CSF cortisol (*F*_(1.4, 20.9)_ = 3.7, *p* = 0.040, η^2^ = 0.039) but not with CSF DHEAS (*F*_(1.4, 8.8)_ = 1.6, *p* = 0.216, η^2^ = 0.017).

When we stratified the analysis by the presence of baseline CI, we found that the interactions between MMSE changes over time and CSF cortisol were not significant in any of the groups. However, the interactions between MMSE changes over time and CSF DHEAS were significant only in the group without CI (*F*_(1.9, 8.5)_ = 4.2, *p* = 0.021, η^2^ = 0.104).

Higher CSF cortisol levels were associated with more pronounced cognitive decline (as evidenced by MMSE score change) and progression in disease severity (as evidenced by CDR-SB score change), while higher CSF DHEAS was only associated with more important progression in disease severity over 36 months.

Cerebrospinal fluid cortisol/DHEAS ratio was not found to be longitudinally associated with the change in MMSE or CDR-SB.

## Discussion

In this prospective study, we found that higher CSF cortisol, but not CSF DHEAS, was associated with poorer baseline cognitive and functional performance. At baseline, CSF cortisol was higher in participants with an AD CSF biomarker profile, in particular in those who also had cognitive impairment. Baseline CSF cortisol and cortisol/DHEAS ratio were associated with CSF tau and p-tau levels, as well as with the baseline volumes of the amygdala and the insula. DHEAS was not associated with any of the AD CSF biomarkers. Furthermore, higher baseline CSF cortisol predicted more pronounced cognitive decline and disease progression over 36 months. Higher baseline CSF DHEAS predicted more pronounced disease progression (as measured by CDR-SB) but not faster MMSE score decline over 36 months.

### Associations Between Cerebrospinal Fluid Cortisol and Dehydroepiandrosterone Sulfate and Baseline Cognitive and Functional Performance

Most previous studies investigating the links between cortisol and cognitive impairment or dementia of AD type used peripheral measures of cortisol, and many reported similar cross-sectional associations between increased cortisol levels (in the plasma, urine, or saliva) and poorer cognitive and functional performance and clinically diagnosed AD (Masera et al., 2002; Armanini et al., 2003; Zverova et al., 2013; Ennis et al., 2017; Ouanes et al., 2017; Ouanes and Popp, 2019). A further study did not find patients with clinically diagnosed AD to have higher CSF cortisol than controls, even though they had higher plasma and urinary cortisol (Pena-Bautista et al., 2019). In a postmortem study, CSF cortisol was higher in the histologically confirmed AD group than in controls, but the difference was only significant in individuals younger than 65 years (Swaab et al., 1994). Intriguingly, in a metabolite profiling study of the CSF in patients with AD, CSF cortisol was found to be the most prominent analyte to separate patients with AD dementia from cognitively healthy controls (Czech et al., 2012). In line with most, but not all previous reports, our findings indicate that higher CSF cortisol is associated with poorer cognitive performance in subjects with cerebral AD pathology and that its levels might be indicators of the severity of the disease (as measured by cognitive performance).

In sum, higher cortisol appears to be cross-sectionally associated with lower cognitive performance, but this association is significant only in subjects with cerebral AD pathology. It is possible that the detrimental effects of cortisol on cognition are only clinically relevant in individuals with developing brain AD pathology. It is also possible that AD pathogenesis itself entails early HPA axis dysfunction. One of the factors leading to this dysfunction might be the early neurodegeneration of structures involved in cognitive performance.

Previous studies found DHEAS levels to be reduced in the CSF and in the brain of AD patients (Kim et al., 2003). Studies which examined plasma DHEAS levels in patients with AD yielded inconsistent results, with some studies finding lower levels among patients with AD (Armanini et al., 2003; Aldred and Mecocci, 2010), and others unable to replicate this finding (Spath-Schwalbe et al., 1990; Legrain et al., 1995). Studies using different biofluids (serum/plasma vs. CSF) may have yielded different results, owing to possible DHEAS synthesis in the brain (Maninger et al., 2009). It is also not clear at which disease stage the decrease in CSF DHEAS might occur. We might have not been able to find an association between CSF DHEAS and cognitive performance, because our most of our participants with cognitive impairment had milder deficits than other studies’ (Legrain et al., 1995; Kim et al., 2003; Aldred and Mecocci, 2010).

Since DHEAS has been shown to counter certain neurotoxic effects of cortisol, cortisol/DHEAS ratio was suggested as a potential marker of glucocorticoid vs. anti-glucocorticoid effects in the brain, and was shown to better distinguish patients with dementia from controls than cortisol or DHEAS alone (Maninger et al., 2009; Hampl and Bicikova, 2010). In the present study, we examined for the first time the association between CSF cortisol/DHEAS ratio and cognitive impairment, and our results were negative. Studies which examined plasma cortisol/DHEAS ratio found the ratio to be increased in patients with dementia (De Bruin et al., 2002), as well as in patients with clinically diagnosed AD type dementia (Masera et al., 2002; Armanini et al., 2003; Magri et al., 2006), with higher ratios being linked to more pronounced cognitive deficit in some (Masera et al., 2002; Magri et al., 2006) but not all (Armanini et al., 2003) studies. This also suggests cortisol/DHEAS ratio in the CSF might have a different significance from the same ratio in plasma, especially since steroids can be synthesized in the brain (Rammouz et al., 2011). The lack of association between CSF DHEAS or cortisol/DHEAS ratio to be associated with cognitive impairment may be explained by the complex relationships between CSF cortisol, DHEAS, and oxidative stress. While DHEAS synthesis in the brain (by conversion from DHEA) may be decreased in AD patients, increased cortisol and oxidative stress can increase DHEA and thus DHEAS levels as a compensatory mechanism (Maninger et al., 2009; Rammouz et al., 2011; Dong and Zheng, 2012).

### Associations Between Baseline Cerebrospinal Fluid Cortisol and Dehydroepiandrosterone Sulfate and Baseline Cerebrospinal Fluid Biomarkers of Alzheimer’s Disease

There is evidence from experimental studies that cortisol might exacerbate Aβ and tau pathology in the brain, while mifepristone, a glucocorticoid antagonist, can decrease cerebral Aβ and tau load in a mouse model of AD (Ouanes and Popp, 2019). In human subjects, one study from the Alzheimer’s Disease Neuroimaging Initiative (ADNI) found CSF cortisol levels to be positively correlated with CSF tau and p-tau, but not CSF Aβ_*1–42*_ (Wang et al., 2018). Findings of studies that measured cortisol levels in the serum have been contradictory (Laske et al., 2009; Pena-Bautista et al., 2019), possibly due to methodological differences and to the relationship between cortisol, Aβ and tau changing as the disease progresses. Indeed, one study in patients with depression found that serum cortisol was not associated with baseline serum Aβ levels, but predicted serum Aβ levels 1 year later (Ishijima et al., 2018). In line with experimental studies our findings suggest that high cortisol in the CNS is involved in AD pathology, in particular in neuronal injury and tau pathology.

We did not observe any associations between CSF DHEAS levels and any of the AD biomarkers, and to the best of our knowledge, there have been no published studies which investigated such links. The relationships between DHEAS and cerebral AD pathology are complex. The presence of Aβ deposits was shown to stimulate the production of DHEA, which is converted to DHEAS in the brain; at the same time, DHEA metabolites might have protective effects against Aβ neurotoxicity and neurodegeneration. There is also some evidence that DHEA conversion to DHEAS might be reduced in AD brains (Maninger et al., 2009).

We found that the cortisol/DHEAS ratio was positively correlated with tau and p-tau. While this is in line with the previously shown detrimental effects of cortisol and protective effects of DHEAS on neurodegeneration (Maninger et al., 2009; Ouanes and Popp, 2019), further studies are needed to clarify the potential role of DHEAS in AD.

### Associations Between Baseline Cerebrospinal Fluid Cortisol and Dehydroepiandrosterone Sulfate and Baseline Brain Volumes

Most previous studies investigating the relationships between cortisol and brain volumes relied on plasma or salivary measures, were cross-sectional, and mostly focused on the hippocampal volumes. Higher salivary or plasma cortisol have been linked to smaller GM volume, and to lower hippocampal and amygdala volume in older community-dwelling individuals without dementia (Sudheimer et al., 2014; Cox et al., 2015; Geerlings et al., 2015), in middle-aged cognitively healthy individuals (Lupien et al., 1998; Echouffo-Tcheugui et al., 2018), as well as in AD (Magri et al., 2006; Huang et al., 2009; Wirth et al., 2019).

The most studied brain structure in its links with cognition, AD pathology, and the HPA axis is the hippocampus. The hippocampus is thought to inhibit the HPA axis. Cortisol can exert detrimental effects on the hippocampus and accelerate its atrophy. The hippocampal atrophy can, in turn, disinhibit the HPA axis, resulting in a vicious circle. This hypothesis, however, was not confirmed by some studies which observed that cortisol levels might be stable over the years in patients with AD (Ouanes et al., 2020).

Amygdala atrophy was shown to be an important imaging finding in early AD. The magnitude of amygdala atrophy was found to be strongly associated with the magnitude of hippocampal atrophy as well as to disease severity. It is also thought to be related to certain neuropsychiatric symptoms in AD including aberrant motor behavior, anxiety and irritability (Poulin et al., 2011). The amygdala is also involved in the HPA axis regulation. Notably, it stimulates the release of corticotropin-releasing hormone (CRH) release. This can explain why the activation of the amygdala has also been shown to be associated with increased cortisol output. This relationship between the amygdala and the HPA axis is implicated in angiogenesis and acute reactions to stress (Hakamata et al., 2017).

Atrophy of the insular cortex may occur at very early, probably asymptomatic, stages in AD. The insula is thought to be involved in neuropsychiatric symptoms, as well as in changes in cardiovascular and autonomic control in AD (Moon et al., 2015). Cortisol seems to increase the functional connectivity between the right anterior insula and medial prefrontal cortex and decrease its functional connectivity with the orbitofrontal cortex (Zhu et al., 2020). This may suggest that that stress-induced cortisol elevations might block the interaction between salience and cognitive processing areas and impede emotion processing. Very few studies investigated the links between DHEAS and brain volumes, and none measured the DHEAS levels in the CSF. Magri et al. (2006) found decreased DHEAS, and increased cortisol/DHEAS ratio in the plasma associated with lower hippocampal volumes in patients with AD dementia.

Taken together, these data indicate a plausible link between elevated cortisol and decreased volume in cortical regions, the amygdala, and the insula. Increased cortisol can enhance neurotoxicity, promote neurodegeneration, and inhibit neuroplasticity and hippocampal neurogenesis through different mechanisms including effects on the brain-derived neurotrophic factor (BDNF) (Suri and Vaidya, 2013), as well as effects on long-term potentiation (Sale et al., 2008), and may therefore contribute to atrophy in vulnerable regions in AD.

### Associations Between Baseline Cerebrospinal Fluid Cortisol and Dehydroepiandrosterone Sulfate, Cognitive Decline and Progression of Disease Severity

We found that higher baseline CSF cortisol independently predicted more marked decline in global and progression of disease severity over 36 months. This confirms the findings of our previous study in an independent cohort where baseline CSF cortisol levels in subjects with AD at the MCI stage predicted faster cognitive decline over time (Popp et al., 2015). Similarly, a study using data from the ADNI reported that higher CSF cortisol levels predicted a more rapid progression from pre-clinical to clinical stages of AD (Udeh-Momoh et al., 2019).

Most studies which investigated the relationship between cortisol levels and cognitive decline over time examined plasma or salivary cortisol. Two previous studies found that higher plasma cortisol levels predicted faster cognitive decline in cognitively healthy older individuals (Pietrzak et al., 2017) as well as in patients with AD (Huang et al., 2009). Studies investigating the links between salivary cortisol and cognitive decline found inconsistent results with associations only present in APOEε4 carriers (Gerritsen et al., 2011), or not at all (Ouanes et al., 2020). These inconsistent results in studies measuring peripheral cortisol levels can reflect that extrapolating plasma or salivary cortisol to brain levels is not always accurate (Martensz et al., 1983). It is also possible that increased cortisol is associated with faster disease progression only at early stages of AD, and that this effect wanes as the disease progresses into later stages. This is supported by our previous findings that baseline CSF cortisol was associated with faster cognitive decline only in the MCI-stage group, and not in the dementia-stage group (Popp et al., 2015).

We found that elevated baseline CSF DHEAS predicted more pronounced progression of disease (as measured by CDR-SB) over 36 months. This finding may seem unexpected given the previously described neuroprotective effects of DHEAS. Indeed, a nested case-control study found lower plasma DHEAS in participants who developed AD 3 years later than in those who did not (Hillen et al., 2000). One possible explanation for our findings might be that DHEAS increases as the disease progresses in response to the increase in cortisol levels in an attempt to regulate its effects on the brain (Maninger et al., 2009; Hampl and Bicikova, 2010). This can also explain why CSF cortisol/DHEAS ratio was not found to be associated with cognitive decline over time in the present study.

The association between CSF cortisol levels and more pronounced progression of AD can have therapeutic implications. Indeed, one may hypothesize that lowering cortisol levels or inhibiting cortisol effects on the brain may help prevent cognitive decline. In this context, several non-pharmacological as well as pharmacological interventions were studied. Even though the initial findings from animal studies were promising for glucocorticoid receptor antagonists or modulators as well as for compounds targeting key enzymes for cortisol synthesis, we are yet to see any convincing evidence from clinical trials (Ouanes and Popp, 2019).

### Strengths and Limitations

We measured central rather than peripheral steroid (cortisol and DHEAS) levels, which better reflects the central nervous system action of these hormones. The free (biologically active) forms of cortisol and DHEAS represent only small fractions in the plasma but are the most prevalent fractions in the CSF (Popp et al., 2015). Correlations between total plasma and CSF levels are generally moderate for steroids, and CSF levels give a much more accurate information about levels that the brain structures are exposed to than mere extrapolation from plasma or salivary levels (Martensz et al., 1983).

This study is, to the best of our knowledge, the first prospective study to examine the relationship between CSF DHEAS and cognitive decline in humans. Moreover, we addressed here for the first time relationships of both CSF cortisol and DHEAS with the “core” AD pathology using well-established CSF biomarkers of amyloid pathology, neuronal injury, and tau pathology. In addition, we assessed relationships with clinical manifestations and disease progression considering measures of both global cognition and disease severity (Morris, 1993). This is also one of very few longitudinal studies examining the link between CSF cortisol and cognitive decline over 2 years.

Nevertheless, a few limitations need to be acknowledged. CSF cortisol and DHEAS levels were measured only at baseline. Hence, we were unable to examine any potential associations between changes in cognition and changes in cortisol and DHEAS levels. In addition, we did not measure saliva or serum/plasma levels of steroids. While these measures can be less accurate than their CSF counterparts, their use in clinical practice might be much easier. Furthermore, brain volumetry was only obtained at baseline, which did not allow us to examine the changes in the structure volumes over time as the cognitive decline occurs. The associations observed at baseline were cross-sectional, which does not allow to identify their direction, let alone to assume causality. As cognitive decline often occurs over a long time span, a longer follow-up period might allow to elicit clinical and biological changes that might be too subtle to detect over 3 years. Last, our choice of analyzing all participants (regardless of their clinical stage) in a single cohort might have led to a heterogenous sample. When we conducted the main analyses for each clinical group separately, our small sample size probably led to type II errors.

## Conclusion

Our findings indicate that higher CSF cortisol levels are involved in the pathophysiology of AD, and are related in particular to neuronal injury and tau pathology, as indicated by CSF tau and p-tau levels, and decreased regional brain volumes. Higher CSF cortisol and DHEAS are associated with more rapid cognitive decline. Modulation of their CNS levels and activity may represent intervention targets to improve symptoms and slow down clinical disease progression.

## Data Availability Statement

The raw data supporting the conclusions of this article will be made available by the authors, without undue reservation.

## Ethics Statement

The studies involving human participants were reviewed and approved by Lausanne University Hospital (Approval No. 171/2013). The patients/participants provided their written informed consent to participate in this study.

## Author Contributions

SO: conceptualization, methodology, data curation, formal analysis, writing–original draft, review and editing, and visualization. CC, PL, JK, and CK: methodology and writing — review and editing. JR and BM: methodology, writing — review and editing and visualization. JP: conceptualization, methodology, data curation, writing — original draft and review and editing, funding acquisition, and supervision. All authors contributed to the article and approved the submitted version.

## Conflict of Interest

JP received consultation honoraria from Nestle Institute of Health Sciences, Innovation Campus, EPFL, Lausanne, Switzerland, Ono Pharmaceutical, OM Pharma and from Fujirebio Europe, all unrelated to the present work. The remaining authors declare that the research was conducted in the absence of any commercial or financial relationships that could be construed as a potential conflict of interest.

## Publisher’s Note

All claims expressed in this article are solely those of the authors and do not necessarily represent those of their affiliated organizations, or those of the publisher, the editors and the reviewers. Any product that may be evaluated in this article, or claim that may be made by its manufacturer, is not guaranteed or endorsed by the publisher.

## Supplementary Material

Supplementary Table 1 Spearman’s bivariate correlations between CSF cortisol and DHEAS levels and brain volumes. CSF, cerebrospinal fluid; DHEAS, dehydroepiandrosterone sulfate; GM, gray matter; WM, white matter. *P*-values were adjusted according to Holm–Bonferroni’s method for *n* = 15 regions of interest. Correlations shown in this table were not adjusted for covariates.
