# Supplementary material for: Cerebrospinal Fluid Cortisol and Dehydroepiandrosterone Sulfate, Alzheimer’s Disease Pathology, and Cognitive Decline
Source: Front Aging Neurosci. 2022 Jul 7;14:892754. doi: 10.3389/fnagi.2022.892754 (PMC9301040; doi:10.3389/fnagi.2022.892754)
Supplement: Supplementary Table 1 — Spearman’s bivariate correlations between CSF cortisol and DHEAS levels and brain volumes. CSF, cerebrospinal fluid; DHEAS, dehydroepiandrosterone sulfate; GM, gray matter; WM, white matter. P-values were adjusted according to Holm–Bonferroni’s method for n = 15 regions of interest. Correlations shown in this table were not adjusted for covariates. [file Table_1.docx]

**Supplementary Table 1: Spearman’s bivariate correlations between CSF cortisol and DHEAS levels and brain volumes**

|  | | | **CSF cortisol** | **CSF DHEAS** | **CSF cortisol/DHEAS** |
| --- | --- | --- | --- | --- | --- |
|  | **GM** | Rho | -.204 | .072 | -.180 |
|  |  | p | .184 | 1.000 | .400 |
|  | **WM** | Rho | -.206 | .022 | -.171 |
|  |  | p | .184 | 1.000 | .400 |
|  | **Frontal GM** | Rho | -.208 | .069 | -.210 |
|  |  | p | .184 | 1.000 | .250 |
|  | **Frontal WM** | Rho | **-.298** | -.009 | -.222 |
|  |  | p | **.014** | 1.000 | .187 |
|  | **Parietal GM** | Rho | -.226 | .047 | -.177 |
|  |  | p | .150 | 1.000 | .400 |
|  | **Parietal WM** | Rho | -.226 | .093 | -.233 |
|  |  | p | .150 | 1.000 | .154 |
| **Temporal GM** | | Rho | **-.294** | .092 | -.252 |
|  |  | p | **.014** | 1.000 | .105 |
| **Temporal WM** | | Rho | -.212 | .012 | -.150 |
|  |  | p | .184 | 1.000 | .444 |
| **Occipital GM** | | Rho | -.160 | .124 | -.193 |
|  |  | p | .348 | 1.000 | .351 |
| **Occipital WM** | | Rho | .117 | .001 | .048 |
|  |  | p | .424 | 1.000 | 1.000 |
| **Hippocampus** | | Rho | -.246 | .028 | -.183 |
|  |  | p | .088 | 1.000 | .400 |
| **Amygdala** | | Rho | **-.300** | .055 | -.237 |
|  |  | p | **.014** | 1.000 | .154 |
| **Insula** | | Rho | **-.350** | .010 | -.236 |
|  | | p | **.000** | 1.000 | .154 |
| **Striatum** | | Rho | -.142 | -.077 | -.064 |
|  | | p | .390 | 1.000 | 1.000 |
| **Thalamus** | | Rho | .111 | -.008 | .040 |
|  | | p | .424 | 1.000 | 1.000 |

*CSF: Cerebrospinal fluid; DHEAS: Dehydroepiandrosterone Sulfate; GM: gray matter; WM: white matter;*

*p values were adjusted according to Holm-Bonferroni’s method for n=15 regions of interest*

*Correlations shown in this table were not adjusted for covariates.*
